# Multiscale modeling for clinical translation in neuropsychiatric disease

**DOI:** 10.1186/2194-3990-1-7

**Published:** 2014-03-03

**Authors:** William W. Lytton, Samuel A. Neymotin, Cliff C. Kerr

**Affiliations:** 1 Department of Physiology & Pharmacology and Neurology, SUNY Downstate Medical Center, Brooklyn, NY 11203, USA; 2 Department of Neurology, Kings County Hospital, Brooklyn, NY 11203, USA

## Abstract

Multiscale modeling of neuropsychiatric illness bridges scales of clinical importance: from the highest scales (presentation of behavioral signs and symptoms), through intermediate scales (clinical testing and surgical intervention), down to the molecular scale of pharmacotherapy. Modeling of brain disease is difficult compared to modeling of other organs, because dysfunction manifests at scales where measurements are rudimentary due both to inadequate access (memory and cognition) and to complexity (behavior). Nonetheless, we can begin to explore these aspects through the use of information-theoretic measures as stand-ins for *meaning* at the top scales. We here describe efforts across five disorders: Parkinson’s, Alzheimer’s, stroke, schizophrenia, and epilepsy. We look at the use of therapeutic brain stimulation to replace lost neural signals, a loss that produces *diaschisis*, defined as activity changes in other brain areas due to missing inputs. These changes may in some cases be compensatory, hence beneficial, but in many cases a primary pathology, whether itself static or dynamic, sets in motion a series of dynamic consequences that produce further pathology. The simulations presented here suggest how diaschisis can be reversed by using a neuroprosthetic signal. Despite having none of the information content of the lost physiological signal, the simplified neuroprosthetic signal can restore a diaschitic area to near-normal patterns of activity. Computer *simulation* thus begins to explain the remarkable success of *stimulation* technologies - deep brain stimulation, transcranial magnetic stimulation, ultrasound stimulation, transcranial direct current stimulation - across an extremely broad range of pathologies. Multiscale modeling can help us to optimize and integrate these neuroprosthetic therapies by taking into consideration effects of different stimulation protocols, combinations of stimulation with neuropharmacological therapy, and interplay of these therapeutic modalities with particular patterns of disease focality, dynamics, and prior therapies.

## Review

Multiscale modeling provides a bridge across scales, from the microscopic to the macroscopic, from the invisible to the visible, from the pharmacological to clinical signs and symptoms. Thus, we can consider multiscale modeling as a way to help connect the two poles of clinical practice: physicians who treat the invisible and surgeons who treat the visible. These bridges are becoming more important in modern practice: the surgeon necessarily utilizes pharmacological treatment, and physicians make ever greater use of interventional techniques to deliver molecules precisely where they are needed. These connections are particularly important in neurosurgical, neurological, and psychiatric disease, where the complexity of the brain makes such scale connections extremely difficult to understand, particularly as one proceeds upward to the realms of cognition, behavior, and memory.

We grossly categorize brain disease as static (fixed lesions) or dynamic (alterations in brain activity patterns). The major dynamical disorders typically have widespread effects: e.g., Alzheimer’s or schizophrenia. However, even a fixed lesion, classically a stroke, does not produce static disease. First, the stroke itself will continue to shrink. Second, other parts of the brain around the lesion will also change, both through diaschisis (strictly defined as alterations due to absent or aberrant brain signals from a damaged area), and through spread of humoral factors. In dealing with brain disease, it would be desirable to restore brain dynamics insofar as possible. In most cases, this dynamic restoration will be imperfect. This is particularly the case in the setting of neurodegenerative disease or in stroke, where there are missing elements that cannot be replaced. However, as we will discuss in this paper, simulation suggests how a lack or loss can be surprisingly well compensated by neuroprostheses that replace only a small part of the lost signals.

Multiscale modeling allows us to move across the scales that are accessed clinically, from the molecular scale of pharmacology up to the whole organism scale where signs and symptoms present. In between, lie most of the scales where our clinical tests assess: both static tests like MRI or CT and dynamic tests like fMRI and electroencephalogram (EEG).

We here give a number of examples where modeling has been used to connect scales in order to better understand two types of complementary interventions: global interventions through pharmacology and localized surgical interventions by electrical stimulation or focal tract ablation. A common theme that we will illustrate is that in many cases the initial brain insult is only the beginning of the pathology; the initial insult sets in motion a series of dynamic consequences that produce further pathology.

### Parkinson’s disease

In Parkinson’s disease (PD), the neurons of the substantia nigra pars compacta of the brainstem degenerate, removing their delivery of dopamine to basal ganglia. This degeneration produces the characteristic tetrad of major symptoms, including slowness of movement and thought (bradykinesia and bradyphrenia, respectively), difficulty coordinating bodily movements (postural instability), rigidity and tremor. Treatment of this disease typically features initial pharmacological intervention. Surgical placement of electrodes for deep brain stimulation is frequently employed later in disease progression.

Initial treatment, using either a dopamine precursor or a dopamine receptor agonist, is sometimes remarkably successful, with the patient reporting a seemingly miraculous return to normal function [1]. This dramatic response is impressive since the original phasic (fast temporal scale) and specific (cellular level spatial scale) has been replaced by continuous (prolonged temporal scale) systemic (brain-wide spatial scale) application. After months to years of treatment, dopamine generally becomes less effective with return of old symptoms and with the emergence of new symptoms which reflect the coexistence of the pathological hypodopaminergic state with an iatrogenic hyperdopaminergic effects characterized by fast choreic movements.

The basal ganglia is an area of substantial complexity. The striatum, the major input area of the basal ganglia, receives highly convergent inputs from the cortical areas. The spiny stellate cells of striatum are GABAergic (inhibitory) neurons that project to the globus pallidus. The globus pallidus is the major output projecting to the thalamus.

Further circuit complexity arises due to the existence of at least two major classes of striatal cells with different types of receptors for dopamine, D1 and D2. These receptors have opposing effects on cell excitability. The cells expressing these receptors are involved in two different types of projections, characterized as a direct and indirect pathway, which have opposing effects. These two circuits have been grossly mapped to two different types of function: a selection circuit and a control circuit. In this hypothesis, a direct pathway from D1 allows release of motor activation at the thalamic level, providing a selection of a specific motor program, while the indirect pathway from D2 striatal cells provides an inhibitory control function at the thalamic level, preventing the release of unwanted motor programs. The tasks that exercise this control/selection balance are switching go/no-go tasks: walk or stand still; stop doing one thing and switch to another; stop thinking about this and think about that. Difficulties with such tasks are characteristic of PD: patients find it hard to initiate a movement or to stop once started (festination).

The basal ganglia is also implicated in learning based on feedback, where dopamine plays a major role in reinforcement learning, associated with dopamine’s producing strong positive feelings when an action leads to positive outcome. Reinforcement learning appears to take place through variation in phasic release of dopamine. By contrast, reduction in dopamine release occurs when an expected reward is not received. These effects are associated with alterations in synaptic plasticity.

Several models of this circuit have been developed. One model looked at the five components of the basal ganglia circuit and provided three competing functional ‘channels’, each representing a particular motor or cognitive task that could be activated preferentially in a sequential action selection paradigm [2]. The model was called upon to select a channel appropriate to an initial stimulus and then switch to a second channel when a second stimulus was presented under conditions of high firing rate (strong activation), indicating that the new stimulus had greater salience. Low levels of dopamine in the model, simulating PD, interfered with both signal selection and switching due to alterations in firing rates associated with the loss of D1 and D2 inputs.

A far different model was utilized to relate activation patterns and spiking outputs to measures of information flow-through in a PD simulation [3]. This model not only crossed different temporal and spatial scales, but also then passed across scales of description - depicting input/output activation relationships utilizing information-theoretic measures. A finding of this study was that information flow, as determined by Granger causality, could show substantial disruption despite relatively unchanged activity by traditional spectral and statistical measures. In these simulations, a neuroprosthetic stimulation not only replaced lost activity but also restored information flow across cortical layers, despite having itself no information content.

The advantage of working with these multiscale models with intermediate levels of network and cellular detail is that they can be utilized to assess different interventional strategies that would be difficult to test in animals or in patients. Interventional strategies could include different electrical stimulation locations and different electrical stimulation patterns, specific pathway or area ablations, or targeted deposit of agonists at particular locations. Pharmacological strategies could include different synergistic drug cocktails, producing combination effects that could not be predicted from the effect of a single drug alone. As noted above, neuroprosthetic intervention and pharmacology are often used together in advanced PD, giving further opportunities to test various mix-and-match options.

### Schizophrenia

In schizophrenia, making the connections to the scale of information is particularly important: this disease manifests in large part as a disorder of thought. Schizophrenia is also a particularly devastating disease, whose impact on society perhaps exceeds those of many later-onset degenerative diseases. Schizophrenia, as a thought disorder, was traditionally handled in a quasi-religious manner, to be cured by replacing wrong-thinking with right-thinking. It is now clear that schizophrenia is a brain disease and that treatments for amelioration or prevention will primarily need to treat the brain disorder, although additional talking treatment may still be valuable to learn behavioral and cognitive compensations.

Schizophrenic patients present with symptoms that are classified into three domains: negative, positive, and cognitive. The cognitive symptoms are considered to be those that most closely reflect the underlying brain dysfunction. Positive and negative symptoms are believed to follow from the central cognitive dysfunction. Positive symptoms include hallucinations (usually auditory) and delusions (false fixed beliefs). Negative symptoms include lack of motivation and social isolation. Cognitive symptoms include deficits of working memory and dysfunction in cognitive coordination, which may be defined in terms of a failure to assess the gestalt of a complete scene or framework. The schizophrenic does not see the whole but instead sees things in a fragmented fashion and may be distracted by irrelevant stimuli which cannot be properly segregated from what is currently pertinent (well described on p. 228 of *The Center Cannot Hold: My Journey to Madness* [4]). Thought is disorganized and the expression of thoughts through speech can become so disjointed that others can no longer follow what the patient is trying to communicate. Because cognitive dysfunction appears to be the core abnormality, cognitive manifestations have been the focus of recent thinking about schizophrenia. Materialists, as opposed to dualists, believe that the mind, responsible for cognition and behavior, is produced directly by brain activity. It therefore follows that neural disco-ordination will underlie cognitive discoordination. One may then seek the connection between the spatial, temporal, and descriptive (informational and meaning) scales of brain activity and the temporal and descriptive scales of thought. Neural discoordination is the hypothesized disorder that affects how different brain regions and brain networks coordinate their functions through shared activity pattern. Symptoms of schizophrenia arise because of failure of neural coordination expressing itself as failure of cognitive coordination.

As in the case of PD, one can relate informational scales based on signals measurable in physical scales of space and time, using information theory to provide numerical estimates of information flow. Consideration of information in the brain raises the question of what codes the brain uses. Rather than treat this question directly here, we will simply consider aspects of some of the many codes that the brain likely uses. These codes, though still not well defined, utilize brain oscillations: oscillations appear to provide the framework for the neural coordination that underlies cognitive coordination [5–7]. These oscillations appear to be abnormally organized in schizophrenia [8–10].

Having now conceptualized linkages downward from cognitive coordination to neural coordination and upward to information, a multiscale model can be constructed to reach down to the relatively low scale of cells and receptors in order to relate neural dis-coordination to potential pharmacological and neuroprosthetic manipulation [11]. Our model focused on the hippocampus, one of the brain structures that appears to be damaged in schizophrenia [12,13]. Multiscale linkage downward to the level of molecules, to neuropharmacology, was made by utilizing an observation from drug abuse: many drugs that block NMDA receptors (one of the classes of glutamate receptor) cause psychotic symptoms resembling those of schizophrenia: delusions, hallucinations, and abnormal thought processes. These *psychotomimetics* include phencyclidine (PCP) and ketamine. Interestingly, these drugs also change brain oscillations, producing an increase in gamma power with concomitant decrease in theta power in mice [14]. Ketamine also produces EEG changes in humans with increase in gamma band oscillations (40 to 85 Hz). These observations connect the pharmacological scale with the network or interareal scale of oscillations.

The existence of these observations at different scales helped determine the scales that needed to be represented in the model: a drug and its receptors (molecular) up through cellular responses, network oscillations, and information flow [11]. At the molecular level, simulated ketamine might alter neurotransmission at any or all of four locations, since different NMDA receptor subtypes are expressed on different cell types and have different sensitivity to a particular NMDA receptor antagonist. These locations were therefore assessed singly and in combination.

**Figure 1:**
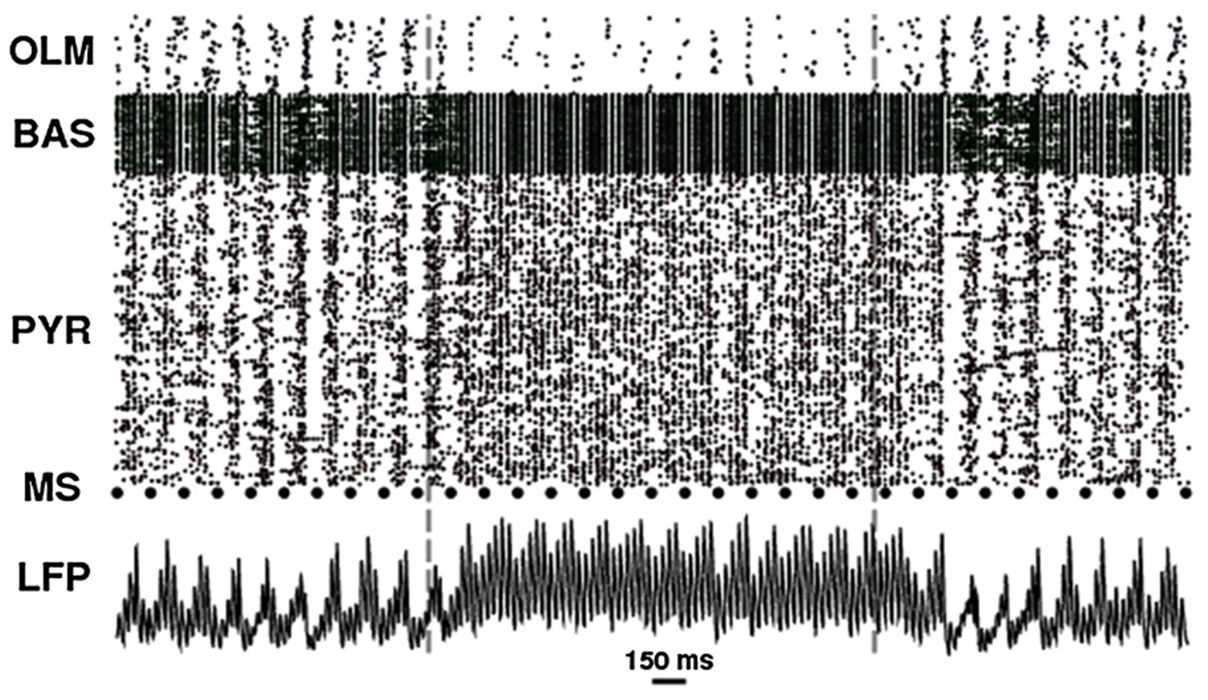
Alterations in spiking and LFP with simulated ketamine application Spike raster (top) and local field potentials (LFP) (bottom) of baseline, ketamine washin, washout. Raster plot at top shows the three types of cells in the model - the ‘OLM’ cells are the ones where the ketamine effect occurs, as can be seen by noting the reduced firing during the period of application. Augmentation of fast oscillation with some reduction in modulation by slow oscillation during the washin is readily seen in the LFP. Vertical dotted lines mark times of washin and washout. Modified with permission from Neymotin et al. [11].

The model was then used to determine site or sites of action of ketamine which would produce effects observed in mouse experiments at the network level: increase in the strength of fast oscillations (gamma) with decrease in strength of slow oscillations (theta). Out of these 16 possible combinations (from four sites), only one replicated the mouse findings - antagonism on one of the types of inhibitory cells. Therefore, it was predicted that the experimental effect of ketamine is based on a primary block at this particular site, connecting the molecular to the network scale (Figure 1).

Information flow was then assessed by looking at nonperiodic signal transmission through this oscillatory network, under both control conditions and under conditions of progressive increase in simulated ketamine dose. Increasing ketamine progressively reduced information flow-through, consistent with the hypothesis that this reduction at the level of information would lead to deficits in complex scene integration, where information integration leads to meaning [15]. (Note that these formal measures of information, based on Shannon’s information theory, do not directly connect to concepts of meaning.)

The association between ketamine’s effect in augmenting fast oscillation and the resultant reduction in information flow-through was an emergent property that was not explicitly built into the model. Although highly speculative, it is helpful to consider the implications of this emergence for behavioral and cognitive manifestations of schizophrenia that otherwise only be handled phenomenologically, detached from pathophysiology. In this context, the conjecture is that the increase in gamma oscillation may represent increased stereotypy of neural activity. Information-theoretically, an oscillation implies a reduced number of ensembles (decreased entropy), reducing representational flexibility.

Jumping to the cognitive realm, such stereotypy/reduced flexibility would reduce thought flexibility and require greater conceptual chunking, leading to repetitive and simplified thought and behavior.

Neuroprosthetic intervention was performed in the model to repair the defect, thereby providing suggestions for therapy. The major pathological effect in the model was a reduction in activation in one of the cell types due to the focal ketamine effect at receptors onto those cells. The treatment was to restore the activity in these cells. In the model, this was done with a continuous activation of the type that could be provided via direct current stimulation. Despite replacing phasic activity with simpler continuous activation, the activation was able to restore activity to a near-normal pattern.

### Epilepsy

Seizures are brief episodes (seconds to minutes) of disturbed brain function due to abnormally prolonged neuronal firing. Along with cardiac arrhythmias, seizures are considered the prototypical dynamical disorder and are therefore the most extensively simulated neurological dysfunction [16]. Epilepsy is the syndrome of recurring seizures. Epilepsy has its own dynamics, much less well understood due to the much longer time scales of years to decades. The dynamics of epilepsy determines when seizures occur, the relations between seizure types, and initialization and development of seizures over years. The causality between individual seizures and epilepsy is one example of how pathology begets pathology: *kindling* occurs as seizures set up other seizure foci leading to more seizures. Kindling is thought to be a pathology due to increased projection activations (the opposite of diaschisis), occurring through use-dependent synaptic plasticity: intense activity will increase synaptic strengths.

Simulation has been used to tie together effects at the molecular scale of abnormal ion channel activation with the abnormal firing (action potential generation) at the cellular level that leads to abnormal prolonged highly synchronous oscillations at the network level. Simulation can assess how an anticonvulsant drug can reduce the likelihood of a seizure without greatly altering the normal patterns of firing. One factor that is often explored is the role of reduced inhibition in causing seizures. Although this can be thought of as a simple excitatory-inhibitory balance whose disruption produces excessive excitability, the role of inhibition is more subtle. In addition to quenching activity, inhibition also plays a major role in synchronization and the setting of oscillatory frequencies where it acts as a pacemaker [17]. This duality produces a number of apparent paradoxes that can be explored through simulation. For example, augmentation of inhibition may cause greater or lesser synchrony in a population depending on the level of inhibition, its time course, and the interaction of inhibition with intrinsic voltage-sensitive channels within the cell [18]. Similarly, the combination of different transmission delays in a feedback system creates a rich repertoire of patterns with different levels of synchrony [19,20].

In a simulation study of the dentate gyrus, an area of the hippocampus, it was noted that reduction of inhibition allowed excitation to spread quickly through a cell population to produce an interictal spike. This single brief population activation effectively used up all of the excitatory cells in a brief and relatively benign activation that then made these cells unavailable, thereby protecting from a seizure [21]. This then suggested the possibility of a surgical strategy employing implanted electrodes: shocking with the correct timing and localization could similarly briefly utilize all of the excitatory cells, leaving them in a simultaneous refractory state that makes them unavailable for involvement in an incipient seizure. This strategy is similar to that which is utilized, and modeled, in forestry, where a prophylactic controlled burn can provide a small area lacking combustibles, which then stops the spread of a wildfire.

The neocortex shows a highly laminar structure with complex connectivity both in depth (across layers) and laterally [22]. The specifics of both depth and lateral projections will differ depending on the specific area of neocortex, with quite different patterns noted in sensory and motor cortices [23]. Ideally, a small ablation could disrupt seizure spread by targeting the particular fiber tracts that are responsible. A recent simulation study evaluated the effects of such microablations or tract sectioning as possible treatment strategies to confine seizures [24]. It demonstrated the interplay between hyperexcitable micronetworks and the connections between them, suggesting that some combination of neuropharmacological quenching of hyperexcitability and surgical ablation might be an optimal strategy.

**Figure 2:**
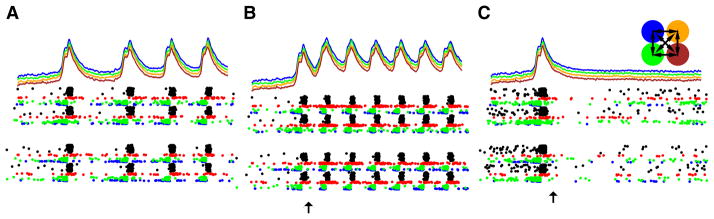
Neuroprosthetic stimulation fails to stop seizure in a neocortical model Four brain areas are shown at the upper right (color coded) with corresponding coloring for local field potentials (LFPs) at the top of each panel. Rasters have a different cell-coded coloring; each trace is 500 ms duration. **(A)** Seizure with activity across all areas nearly simultaneous at 12 Hz. **(B)** Attempted seizure abort with one area of stimulation fails, instead creating a faster seizure (16 Hz). **(C)** Seizure aborted with activation of inhibitory cells alone. From Lytton et al. [24].

This study also revisited the ‘controlled burn’ approach to seizure occlusion, utilizing a high-amplitude stimulation to activate a set of excitatory cells. In this case, the technique failed, illustrating that details are critical in multiscale modeling: the same cause will have entirely different effects depending on hidden details of wiring or dynamics. Figure 2 demonstrates the failure in this neocortical model of a method that worked in a dentate gyrus model. Regardless of the phase or strength of stimulation of the control simulation (Figure 2A), the coherent epileptiform oscillation continues to spread across the four areas (upper right inset) being simulated. Post-stimulation activity for a particular example of stimulation shows an increase in frequency from 12 to 16 Hz (Figure 2B). The study determined a number of factors responsible for this failure, both involving discrepancies in scale between the treatment (brief in time and narrow spatially) and aspects of the system being treated (prolonged in time and broad in spatial extent). The spatial scale of intercolumnar projections made it difficult to involve enough cells in the stimulation to give efficacy (note that we did not want to simply stimulate all of the cells since the idea was to demonstrate that a focal stimulation could prevent or abort a more widespread seizure). Additionally, the prolonged temporal scale of NMDA activation provided ongoing activation in cell dendrites that could outlast the effects of the stimulation.

Further exploration of this simulation did provide a clue to a stimulation technique that might be more effective: stimulation of only the inhibitory cells provided sufficiently strong, sufficiently prolonged, and sufficiently widespread inactivation to break the pattern of oscillatory spread across the columns (Figure 2C). Such stimulation could be effected therapeutically through focal alterations of inhibitory cells in a small area of cortex so as to make them susceptible to local light stimulation (optogenetics) with an implanted light source. Alternatively, one could use a more global stimulation of central projections with widespread projections (e.g., brainstem areas that provide and project neuromodulators broadly to cortex) to activate inhibitory cells, perhaps requiring partial pharmacological blockade to reduce neuromodulatory effects on the excitatory population.

### Stroke and diaschisis

The brain requires a continuous supply of nutrients. Interruption rapidly leads to cell death. The process of stroke and stroke recovery can be modeled at many scales, from the molecular processes operating in the cell that lead to damage, to the spread of that damage due to toxic factors, to the recovery of function through compensation for lost brain tissue [25]. In stroke, pathological dynamics begets pathological dynamics through diaschisis: a reduction in activity in remote areas of the brain due to the absence of activation that had been received from the now missing area [26]. These same perturbations, with somewhat different scale, also occur following traumatic brain injury. In this way, stroke or trauma can have far-reaching effects in the brain [27].

Projections between brain areas transfer both activation and information. In the context of signals and systems, this activation can be regarded as involving the carrier signal. There is much evidence to suggest that the carrier signals in the brain, as in analog radio transmission, are oscillatory. As we have described above and will again show here, this dual nature of brain signaling can be utilized to replace specific signals with nonspecific signals.

There is a hierarchy of neocortical processing that involves projections upward (both direct and via thalamus) from primary sensory areas to multiple higher areas [28]. These higher areas are involved in associating aspects to form a gestalt perception (through neural coordination) and in making predictions. The coordinating of present information and of future predictions (a coordination through time) are then fed back to lower areas in order to signal how primary sense inputs should be organized and what features of primary sense inputs should be ignored [29].

**Figure 3:**
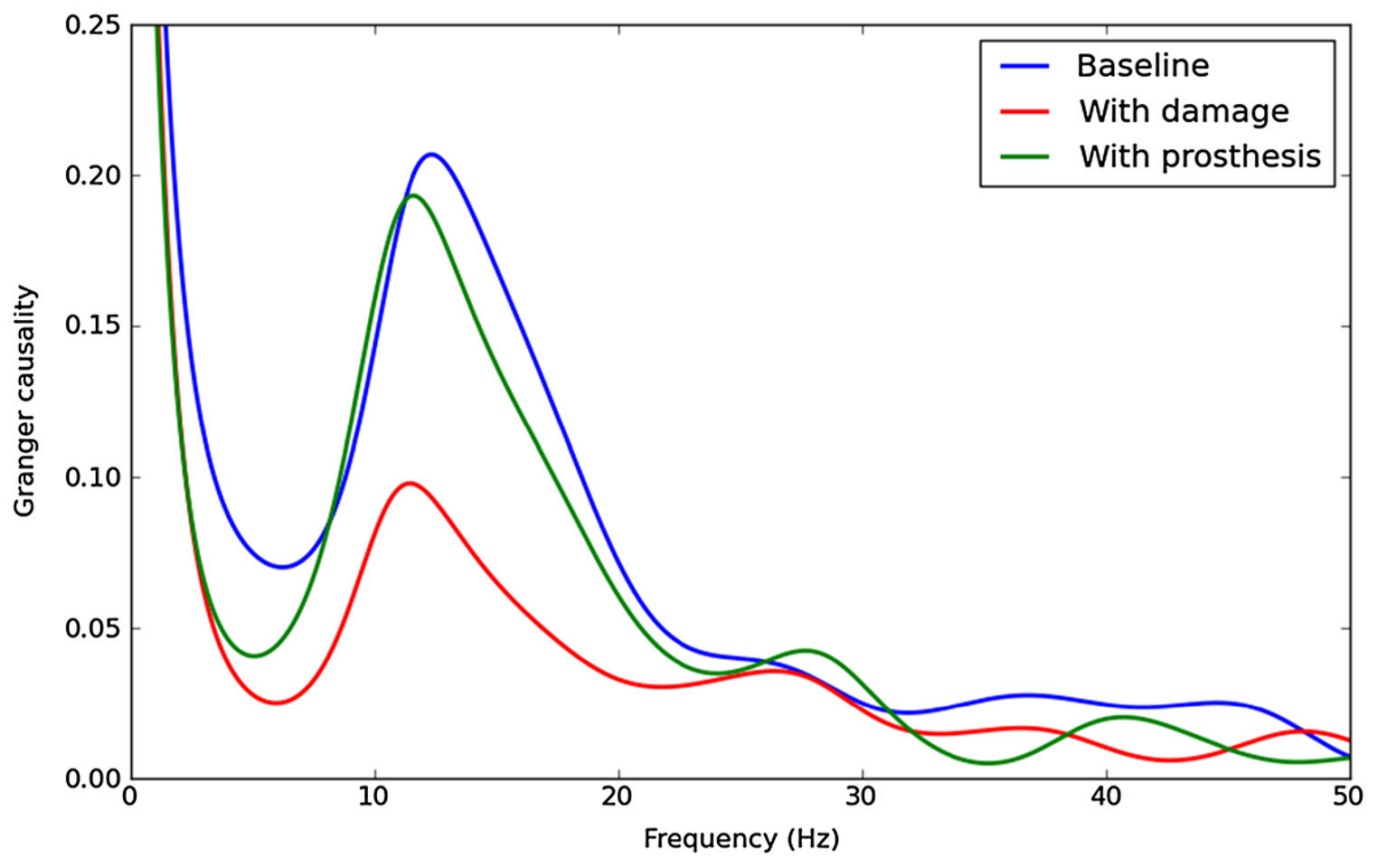
Recovery of information flow with neuroprosthetic stimulation Spectral Granger causality between layers of cortex shows recovery of information flow-through after application of neuroprosthetic signal: baseline (blue), diaschisis with absent inputs (red), after prosthetic stimulation (green). From Kerr et al. [30].

A model of diaschisis repair was developed which assessed the effects of a neuroprosthesis on an area of association cortex [30]. The replacement was with a sinusoidal signal in the 10 Hz (alpha) range. As expected, this additional activation largely normalized dynamics as measured by rates and local field potential spectrum. More interestingly, the stimulation also recovered information flow between layers as measured by Granger causality (Figure 3). As in the Parkinson’s disease example, the neuroprosthetic signal, despite lacking information content, restored much of the information quantity between layers in the intact cortical area that had been damaged by diaschisis. Note that we are only demonstrating information quantity here and that the information quality (meaning), not readily measurable, would not be fully restored, since it is critically dependent on the specifics of the now-missing inputs.

### Alzheimer’s disease

There is still considerable debate about the underlying dysfunction that leads to the degeneration of cells and synapses in Alzheimer’s disease. As with the other diseases we have discussed, this initial damage is followed by a set of dynamic shifts that may in some cases serve as compensations but in other cases will cause further damage [31]. One hypothesis suggests that initial cell death and synapse dysfunction are a consequence of a natural homeostatic response at the cellular level [32]. As cells die, remaining cells lose their inputs (*cf.* diaschisis). These remaining cells suffer an initial reduction in activity, followed by cell-level homeostatic synaptic scaling which begins to restore cellular activity [33].

The homeostatic mechanism involves calcium levels and other indicators of firing activity and synaptic activity. Synaptic scaling will increase synaptic strength from remaining presynaptic projections onto a surviving cell. This synaptic scaling will then increase drive and restore activity. However, stronger and fewer synapses provides a pathological substrate: higher connection gain means that small fluctuations are amplified, leading to periods of overdrive that can cause additional cell death through excitotoxicity (molecular and cell level pathology). In some cases, these fluctuations will lead to localized network bursting responses due to spread of activity in this smaller, more vulnerable, circuit (network level pathology) [34,35]. This abnormal firing, a miniature seizure in the miniature circuit, is likely to interfere with remaining cognitive resources. Such miniature seizures can also become full-fledged seizures.

Similar to the prior example of stroke and diaschisis, a simulation study was performed to evaluate the ability of neuroprosthetic stimulation to interfere with this process by putting back the missing signal [36]. This intervention eliminated the underlying cause of homeostatic synaptic scaling by driving the cells so as to move them back to their characteristic activity level. This study also demonstrated that such a neuroprosthesis would not interfere substantially with information flow, reducing concerns that the therapy might damage the ability of the network to incorporate new learning.

A number of clinical studies have suggested the efficacy of deep brain stimulation (DBS), transcranial magnetic stimulation (TMS), or transcranial direct current stimulation (tDCS) to slow the progression of AD [37,38]. The modeling here provides explanation and exploration as to how, why, and when such stimulation protocols might be efficacious.

## Conclusions

A recurring theme in this set of studies has been that pathological dynamics, often involving initial abnormality in signal transmission, may lead to secondary compensatory signal abnormalities whose pathological impact may be as great or greater than the initial insult.

A major gap in our understanding of the brain activity is the difficulty of relating information theoretic measures, which measure statistical properties of signals, to the level of dysfunction in meaning and thought, a critical feature of schizophrenia and other disorders. In addition to demonstrating the flow of information, simulations can contribute to filling this gap as they are called upon to produce behaviors: converting a signal to a meaningful output or perception - interpreting complex stimulus sets by extracting meaning from a jumble of inputs [39–43]. This is the notion of *embodiment*, the idea that the functioning of the neural systems depends on the continuing feedback from the body and the world. In this view, neural functioning cannot be adequately modeled as an isolated system - the world being another scale in the multiscale modeling approach.

These simulation studies suggest how highly simplified signals can be used to replace the complex physiological signals that are lost in a variety of neuropsychiatric disorders. We have shown in a number of cases how replacement of the signal might have a therapeutic value, despite lack of signal structure. This may in part explain the unusual success of transcranial direct current stimulation, among other stimulation modalities, in a remarkably wide array of disorders, including those explored in this review [44].

## References

[1] Sachs O (1983). Awakenings.

[2] Humphries MD, Stewart RD, Gurney KN (2006). A physiologically plausible model of action selection and oscillatory activity in the basal ganglia. J Neurosci.

[3] Kerr CC, van Albada SJ, Neymotin SA, Chadderdon GL, Robinson PA, Lytton WW (2013). Cortical information flow in Parkinson’s disease: a composite network/field model. Front Comput Neurosci.

[4] Saks ER (2008). The Center Cannot Hold: My Journey Through Madness.

[5] Engel A, Konig P, Kreiter A, Gray C, Singer W, Schuster HG (1991). Temporal coding by coherent oscillations as a potential solution to the binding problem: physiological evidence. Nonlinear Dynamics and Neural Networks.

[6] Gray C, Singer W (1989). Stimulus-specific neuronal oscillations in orientation columns of cat visual cortex. Proc Nat Acad Sci.

[7] Gray CM, König P, Engel AK, Singer W (1989). Oscillatory responses in cat visual cortex exhibit inter-columnar synchronization which reflects global stimulus properties. Nature.

[8] Uhlhaas PJ, Linden DE, Singer W, Haenschel C, Lindner M, Maurer K, Rodriguez E (2006). Dysfunctional long-range coordination of neural activity during gestalt perception in schizophrenia. J Neurosci.

[9] Uhlhaas PJ, Singer W (2006). Neural synchrony in brain disorders: relevance for cognitive dysfunctions and pathophysiology. Neuron.

[10] Uhlhaas PJ, Singer W (2007). What do disturbances in neural synchrony tell us about autism. Biol Psychiatry.

[11] Neymotin SA, Lazarewicz MT, Sherif M, Contreras D, Finkel LH, Lytton WW (2011). Ketamine disrupts theta modulation of gamma in a computer model of hippocampus. J Neurosci.

[12] Harrison PJ (2004). The hippocampus in schizophrenia: a review of the neuropathological evidence and its pathophysiological implications. Psychopharmacology.

[13] Tseng KY, Chambers RA, Lipska BK (2008). The neonatal ventral hippocampal lesion as a heuristic neurodevelopmental model of schizophrenia. Behav Brain Res.

[14] Lazarewicz MT, Ehrlichman RS, Maxwell CR, Gandal MJ, Finkel LH, Siegel SJ (2010). Ketamine modulates theta and gamma oscillations. J Cogn Neurosci.

[15] Tononi G, Edelman GM (1998). Consciousness and complexity. Science.

[16] Lytton WW (2008). Computer modelling of epilepsy. Nat Rev Neurosci.

[17] Lytton WW, Sejnowski TJ (1991). Simulations of cortical pyramidal neurons synchronized by inhibitory interneurons. J Neurophysiol.

[18] Deyo S, Lytton WW (1997). Inhibition can disrupt hypersynchrony in model neuronal networks. Prog Neuropsychopharmacol Biol Psychiatry.

[19] Brunel N, Hakim V (1999). Fast global oscillations in networks of integrate-and-fire neurons with low firing rates. Neural Comput.

[20] Sirovich L, Omurtag A, Lubliner K (2006). Dynamics of neural populations: stability and synchrony. Network.

[21] Lytton WW, Hellman KM, Sutula TP (1996). Computer network model of mossy fiber sprouting in dentate gyrus. Epilepsia – AES Proc.

[22] Neymotin SA, Lee H, Park E, Fenton AA, Lytton WW (2011). Emergence of physiological oscillation frequencies in a computer model of neocortex. Front Comput Neurosci.

[23] Hooks BM, Hires SA, Zhang YX, Huber D, Petreanu L, Svoboda K, Shepherd GM (2011). Laminar analysis of excitatory local circuits in vibrissal motor and sensory cortical areas. Plos Biol.

[24] Lytton WW, Neymotin SA, Wester JC, Contreras D, Bass B, Garbey M (2013). Neocortical simulation for epilepsy surgery guidance: localization and intervention. Computational Surgery and Dual Training.

[25] Lytton WW, Williams ST, Sober SJ (1999). Unmasking unmasked: neural dynamics following stroke. Prog Brain Res.

[26] von Monakow C (1914). Die Lokalisation im Grosshirn und der Abbau der Funktion durch kortikale Herde.

[27] Meyer JS, Obara K, Muramatsu K (1993). Diaschisis. Neurol Res.

[28] Van Essen D, Anderson CH, Felleman DJ (1992). Information processing in the primate visual system: an integrated systems perspective. Science.

[29] Bastos AM, Usrey WM, Adams RA, Mangun GR, Fries P, Friston KJ (2012). Canonical microcircuits for predictive coding. Neuron.

[30] Kerr CC, Neymotin SA, Chadderdon GL, Fietkiewicz CT, Francis JT, Lytton WW (2012). Electrostimulation as a prosthesis for repair of information flow in a computer model of neocortex. IEEE Trans Neural Syst Rehab Eng.

[31] Small DH (2008). Network dysfunction in Alzheimer’s disease: does synaptic scaling drive disease progression?. Trends Mol Med.

[32] Turrigiano GG (2008). The self-tuning neuron: synaptic scaling of excitatory synapses. Cell.

[33] van Rossum MCW, Bi GQ, Turrigiano GG (2000). Stable Hebbian learning from spike timing-dependent plasticity. J Neurosci.

[34] Busche MA, Eichhoff G, Adelsberger H, Abramowski D, Wiederhold KH, Haass C, Staufenbiel M, Konnerth A, Garaschuk O (2008). Clusters of hyperactive neurons near amyloid plaques in a mouse model of Alzheimer’s disease. Sci Signall.

[35] Fröhlich F, Bazhenov M, Sejnowski TJ (2008). Pathological effect of homeostatic synaptic scaling on network dynamics in diseases of the cortex. J Neurosci.

[36] Rowan M, Neymotin S (2013). Synaptic scaling balances learning in a spiking model of neocortex. Springer LNCS.

[37] Hansen N (2012). Action mechanisms of transcranial direct current stimulation in Alzheimer’s disease and memory loss. Front Psychiatry.

[38] Smith GS, Laxton AW, Tang-Wai DF, McAndrews MP, Diaconescu AO, Workman CI, Lozano AM (2012). Increased cerebral metabolism after 1 year of deep brain stimulation in Alzheimer disease. Arch Neurol.

[39] Chadderdon GL, Neymotin SA, Kerr CC, Lytton WW (2012). Reinforcement learning of targeted movement in a spiking neuronal model of motor cortex. PLOS ONE.

[40] Dura-Bernal S, Chadderdon GL, Neymotin SA, Francis JT, Lytton WW (2014). Towards a real-time interface between a biomimetic model of sensorimotor cortex and a robotic arm. Pattern Recognit Lett.

[41] Neymotin SA, Chadderdon GL, Kerr CC, Francis JT, Lytton WW (2013). Reinforcement learning of 2-joint virtual arm reaching in computer model of sensorimotor cortex. Neural Comput.

[42] Song W, Kerr CC, Lytton WW, Francis JT (2013). Cortical plasticity induced by spike-triggered microstimulation in primate somatosensory cortex. Plos One.

[43] Neymotin SA, Jacobs KM, Fenton AA, Lytton WW (2011). Synaptic information transfer in computer models of neocortical columns. J Comput Neurosci.

[44] Brunoni AR, Nitsche MA, Bolognini N, Bikson M, Wagner T, Merabet L, Edwards DJ, Valero-Cabre A, Pascual-Leone A, Ferrucci R, Priori A, Boggio PS, Fregni F (2012). Clinical research with transcranial direct current stimulation (tDCS): challenges and future directions. Brain Stimul.

